# Identification and Optimization of Classifier Genes from Multi-Class Earthworm Microarray Dataset

**DOI:** 10.1371/journal.pone.0013715

**Published:** 2010-10-28

**Authors:** Ying Li, Nan Wang, Edward J. Perkins, Chaoyang Zhang, Ping Gong

**Affiliations:** 1 School of Computing, University of Southern Mississippi, Hattiesburg, Mississippi, United States of America; 2 Environmental Laboratory, U.S. Army Engineer Research and Development Center, Vicksburg, Mississippi, United States of America; 3 Environmental Services, SpecPro Inc., Vicksburg, Mississippi, United States of America; Michigan State University, United States of America

## Abstract

Monitoring, assessment and prediction of environmental risks that chemicals pose demand rapid and accurate diagnostic assays. A variety of toxicological effects have been associated with explosive compounds TNT and RDX. One important goal of microarray experiments is to discover novel biomarkers for toxicity evaluation. We have developed an earthworm microarray containing 15,208 unique oligo probes and have used it to profile gene expression in 248 earthworms exposed to TNT, RDX or neither. We assembled a new machine learning pipeline consisting of several well-established feature filtering/selection and classification techniques to analyze the 248-array dataset in order to construct classifier models that can separate earthworm samples into three groups: control, TNT-treated, and RDX-treated. First, a total of 869 genes differentially expressed in response to TNT or RDX exposure were identified using a univariate statistical algorithm of class comparison. Then, decision tree-based algorithms were applied to select a subset of 354 classifier genes, which were ranked by their overall weight of significance. A multiclass support vector machine (MC-SVM) method and an unsupervised K-mean clustering method were applied to independently refine the classifier, producing a smaller subset of 39 and 30 classifier genes, separately, with 11 common genes being potential biomarkers. The combined 58 genes were considered the refined subset and used to build MC-SVM and clustering models with classification accuracy of 83.5% and 56.9%, respectively. This study demonstrates that the machine learning approach can be used to identify and optimize a small subset of classifier/biomarker genes from high dimensional datasets and generate classification models of acceptable precision for multiple classes.

## Introduction

DNA microarray, a maturing genomic technology, has been used extensively as a diagnostic tool to complement traditional approaches such as histopathological examination for various diseases (particularly cancers) because microscopic appearances sometimes can be deceiving [1]–[4]. Microarrays have also successfully served as a research tool in discovering novel drug targets [5] and disease- or toxicity-related biomarker genes for cancer classification [6]. In ecological risk assessment, indigenous species such as fish and earthworms are often used as bioindicators for adverse effects caused by environmental contaminants. Previously, we developed an earthworm (*Eisenia fetida*) cDNA microarray to analyze toxicological mechanisms for two military-unique explosive compounds 2,4,6-trinitrotolune (TNT) and 1,3,5-trinitro-1,3,5-triazacyclohexane (also known as Royal Demolition eXplosive or RDX) [7], [8]. These two compounds exhibit distinctive toxicological properties that are accompanied by significantly different gene expression profiles in the earthworm *E. fetida* [7]–[9], which has motivated us to look further into toxicant- or toxicity-specific signature genes/biomarkers. The second motivation comes from the fact that many diagnostic assays exist for human diseases while very few are available for evaluating impacts on environmentally-relevant organisms. Gross survival, growth and reproduction rates are often used as assessment endpoints without reflecting the diseased population of affected animals that is an important part of long-term impact assessment. The last motivation is that computational tools such as machine learning techniques have been widely used in cancer and toxicant classification with microarray data but rarely applied in microarray data analysis of environmentally relevant organisms [10]–[12].

From a regulatory standpoint, there is an increasing and continuous demand for more rapid, more accurate and more predictive assays due to the already large, but still growing, number of man-made chemicals released into the environment [13]. Molecular endpoints such as gene expression that may reflect phenotypic disease symptoms manifested later at higher biological levels (e.g., cell, tissue, organ, or organism) are potentially biomarkers that meet such demands. As a high throughput tool, microarrays simultaneously measure thousands of biologically-relevant endpoints (gene expression). However, to apply this tool to animals under field conditions, one critical hurdle to overcome is the separation of toxicity-induced signals from background noise associated with environmental variation and other confounding factors such as animal age, genetic make-up, physiological state and exposure length and route [10], [11]. A common approach to biomarker discovery is to screen genome- or transcriptome-wide gene expression responses and identify a small subset of genes capable of discriminating animals that received different treatments, or predicting the class of unknown samples. It is relatively less challenging to identify differentially expressed genes from two or more classes of samples. However, the search for an optimal and small subset of genes that has a high discriminatory power in classifying field samples often having multiple classes is much more complicated.

For instance, Falciani and colleagues profiled gene expression of 77 hepatic samples of European flounder (*Platichthys flesus*) collected from six different environmental sites [10]. Using a multivariate variable selection coupled with a statistical modelling procedure they demonstrated that the accuracy of predicting the geographical site of origin based on gene expression signatures in flounder livers was limited to specific sites. After incorporating prior knowledge and data from laboratory exposures to individual toxicants, they were able to limit the search space for a combination of effective classifier genes and built a very accurate model consisting of only 17 genes for classification of all the different environmental sites. Similarly, Nota and co-workers recently identified a set of 188 genes from expression profiles of the springtail (*Folsomia candida*) exposed to a soil spiked with six different metals using the uncorrelated shrunken centroid method, and predicted an independent test soils set with an accuracy of 83% but failed on field soils collected from two cobalt-contaminated sites using this gene set [11]. Several other studies also reported a varying degree of success in the identification of classifier genes in both aquatic species like the zebrafish (*Danio rerio*) [12], the common carp *Cyprinus carpio* [14] and the water flea *Daphnia magna* [15], and terrestrial organisms such as the earthworm *Lumbricus rubellus* [16].

As part of a larger effort towards discovering novel biomarkers for ecological risk assessment of military lands, we have developed a 15208-oligonucleotide *E. fetida* array, and generated a large-scale microarray dataset from a laboratory study where earthworms (*E. fetida*) were exposed to various concentrations of TNT or RDX for various lengths of time in soil, mimicking field exposure scenarios. The objective of the current study was to identify a small set of classifier genes that could be used to build a predictive model capable of accurately separating all exposed earthworm samples into three categories: control, TNT-treated and RDX-treated. We focused on identifying and optimizing classifier genes from the earthworm dataset using a machine learning approach.

## Materials and Methods

### Experimental design and dataset generation

Adult earthworms (*E. fetida*) were exposed in a field collected pristine silty loam soil (3% sand, 72% silt, 26% clay, pH 6.7, total organic C 0.7%, and CEC 10.8 mEq/100 g) spiked with TNT (0, 6, 12, 24, 48, or 96 mg/kg) or RDX (8, 16, 32, 64, or 128 mg/kg) for 4 or 14 days. The 4-day treatment was repeated a second time with the same TNT concentrations, however RDX concentrations were 2, 4, 8, 16 or 32 mg/kg soil. Each treatment originally had 10 replicate worms with 8∼10 survivors at the end of exposure, except the two highest TNT concentrations. At 96 mg TNT/kg, no worms survived in the original 4-day and 14-day exposures, whereas at 48 mg TNT/kg, all 10 worms died in the original 4-day exposure. Total RNA was isolated from the surviving worms as well as the Day 0 worms (worms sampled immediately before experiments). A total of 248 worm RNA samples ( = 8 replicate worms×31 treatments) were hybridized to a custom-designed oligo array using Agilent's one-colour Low RNA Input Linear Amplification Kit. The array contained 15,208 non-redundant 60-mer probes (GEO platform accession number GPL9420), each targeting a unique *E. fetida* transcript [17]. After hybridization and scanning, gene expression data were acquired using Agilent's Feature Extraction Software (v.9.1.3). In the current study, the 248-array dataset was divided into three worm groups regardless of exposure length and concentraiton: 32 untreated controls, 96 TNT-treated, and 120 RDX-treated. This MIAME compliant dataset has been deposited in NCBI's Gene Expression Omnibus [18] and is accessible through GEO Series accession number GSE18495 (http://www.ncbi.nlm.nih.gov/geo/query/acc.cgi?acc=GSE18495).

### Integrated Statistical and Machine Learning (ISML) approach

**Figure 1:**
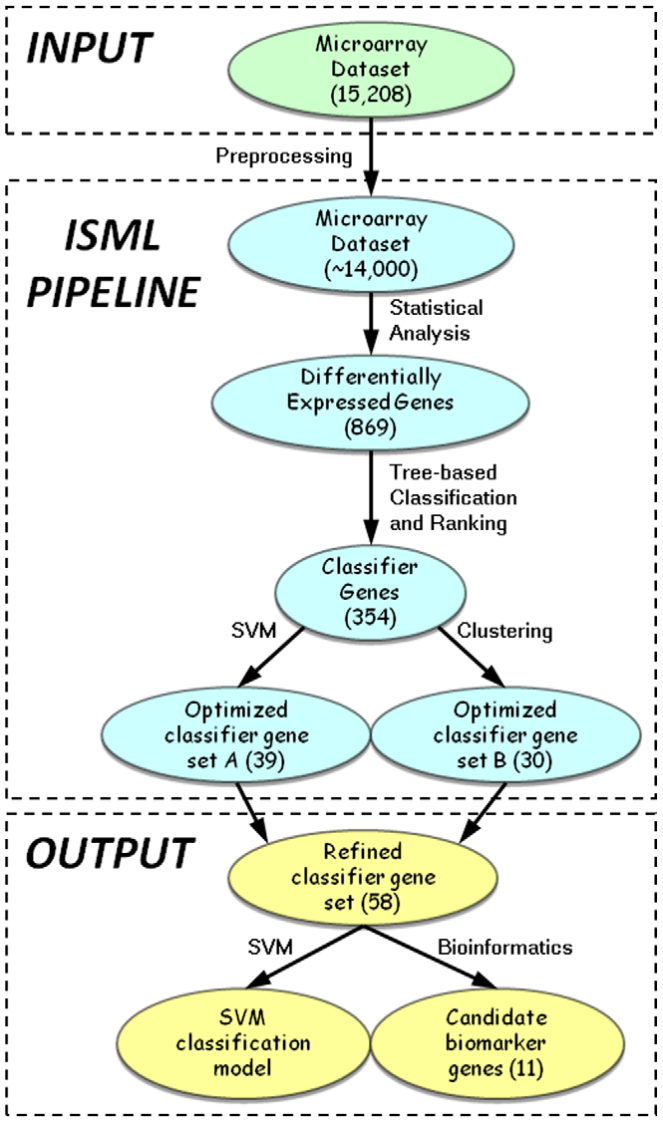
Overview of the integrated statistical and machine learning (ISML) pipeline. The pipeline illustrates the analytical procedure that integrates statistical analysis with supervised machine learning and unsupervised clustering as described in **Materials and Methods**. Numbers in brackets indicate the amount of genes remaining (also see **Results**).

A challenge in classifying or predicting the diagnostic categories using microarray data is the curse of dimensionality problem coupled with sparse sampling. That is, the number of examined genes per sample is much greater than the number of samples that are involved in classification [19]. The other crucial challenge is that the huge search space for an optimal combination of classifier genes renders high computational expenses [20]. To address these two issues, we developed the new ISML pipeline, which integrates statistical analysis with supervised and unsupervised machine learning techniques (Fig. 1). The pipeline consists of four major components: (1) statistical analysis that reduces dimensionality through identification of the most differentially expressed genes; (2) tree-based algorithms that are used to further downsize the number of classifier genes with assigned weight and associated ranking; (3) MC-SVM and unsupervised clustering, each of which independently selects an optimal set of classifier genes using an iterative elimination process (see **Optimization of classifier genes by MC-SVM** below for details); and (4) the integration of the two independent gene sets to generate a final refined gene sets.

### Data pre-processing

The following data pre-treatment steps were applied prior to further statistical and computational analyses: (1) feature filtering: flag out spots with signal intensity outside the linear range as well as non-uniform spots; (2) conversion: convert signal intensity into relative RNA concentration based on the linear standard curve of spike-in RNAs; (3) normalization: normalize the relative RNA concentration to the median value on each array; and (4) gene filtering: filter out genes appearing in less than 50% of arrays (i.e., present on at least 124 arrays). There were more than 14,000 genes remaining after this procedure.

### Feature filtering by univariate statistical analysis

The Class Comparison Between Groups of Arrays Tool in BRB-ArrayTools v.3.8 software package ([21]; linus.nci.nih.gov/BRB-ArrayTools.html) was used to identify significantly changed genes. The collated earthworm array dataset was imported without any further normalization or transformation. The tool runs a random variance version of the t-test or F-test separately for each gene. It performs random permutations of the class labels and computes the proportion of the random permutations that give as many genes significant at the level set by the user as are found in comparing the true class labels. The following eight class-comparison analyses were conducted to infer genes differentially expressed in response to TNT or RDX: (1) two 2-class comparisons: pooled controls vs. pooled TNT or RDX treatments; and (2) six multiple-class comparisons: 4-day TNT or RDX multiple concentrations, 4-day repeat TNT or RDX multiple concentrations, and 14-day TNT or RDX multiple concentrations. The following settings were employed: a univariate test random variance model, multivariate permutation tests with 10,000 random permutations, a confidence level of false discovery rate assessment = 99%, and a maximum allowed number of false-positive genes = 10.

### Classifier gene selection by tree-based algorithms

Seven decision tree methods (SimpleCart, BFTree, FT, J48, LADTree, LMT and REPTree) were used for gene selection to avoid the biases and overcome limitations of each single algorithm [22], [23]. An ensemble strategy was also applied to increase prediction accuracy using bagging (Bagging) and boosting (AdaBoostM1) [24]. All of these algorithms are implemented in the WEKA machine learning workbench v.3.6.0 ([25]; www.cs.waikato.ac.nz/ml/weka/). The resulting tree structure each generated a set of classifier genes. The performance of a classifier was evaluated using three criteria: accuracy (see below for definition), precision (or sensitivity = number of correctly classified samples/total number of samples classified into this class), and the area under the ROC (Receiver Operating Characteristic) curve.

### Ranking classifier genes by weight of significance

A weight of significance was assigned on a scale between 0 and 1 to every selected classifier gene based on its position/significance in an assembled decision tree according to Equation (1):
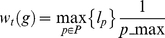
(1)where 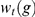 is the weight of gene 
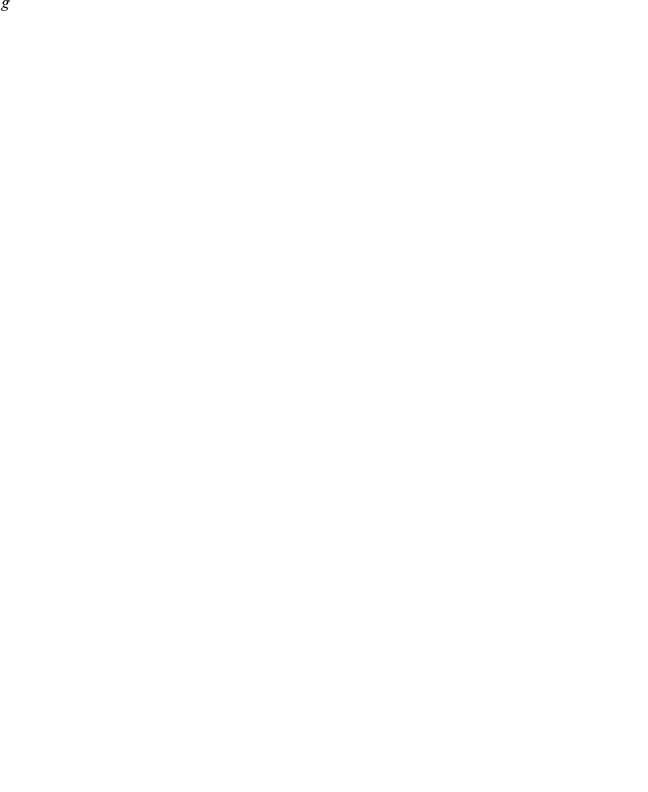
 assigned by a tree model 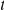, 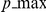 is the longest path of the tree, and 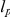 is the height of the gene in path 
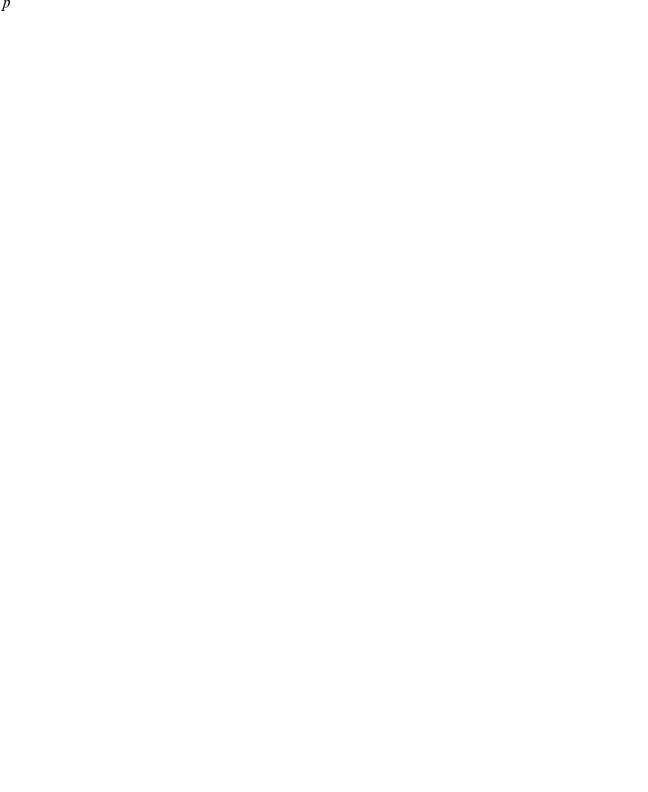
. A “root” gene was awarded the largest weight whereas a “leaf” gene the smallest. The weight value was normalized to the longest leaf-to-root path, except for those genes selected by the LMT algorithm, whose weight had already been assigned by a logistic model. The overall weight for a classifier gene, i.e., the sum of its weight assigned in all the decision tree methods, was calculated as follows:
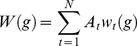
(2)where 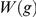 is the overall weight of gene 
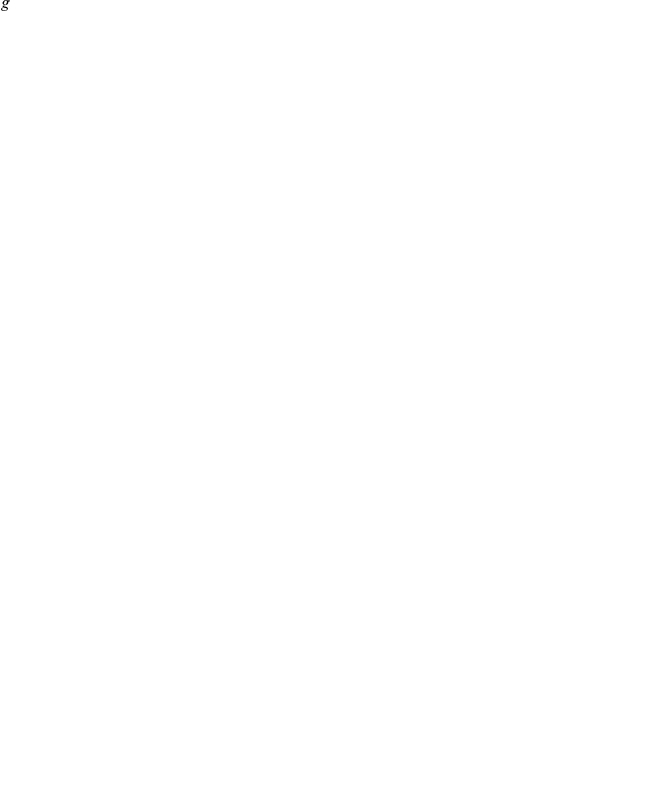
, 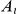 is the accuracy of tree model 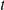, and *N* is the total number of tree models. All of the classifier genes were ranked by their overall weight, i.e., the larger the weight it had, the higher it ranked.

### Optimization of classifier genes by MC-SVM

Sequential minimum optimization (SMO), a fast algorithm for training SVM [26], [27], was used to build MC-SVM kernel function models, as implemented in WEKA. We designed the following steps to refine the classifier gene set:

1. start with the highest ranking classifier gene to train the SVM using the training dataset and classify the testing dataset using the trained SVM;
2. add one gene of immediately lower ranking in overall weight at a time to constitute a new gene set, and use the gene set to train and predict the samples; repeat this step until all the classifier genes have been included;
3. calculate the classification accuracy of each class (control, TNT and RDX) and the weighted average accuracy of all three classes for each set of genes using results from the testing dataset;
4. estimate the improvement or decline in classification accuracy as a result of adding one gene for each of the three classes plus the weighted average accuracy of all three classes;
5. remove any gene(s) starting from the one ranking at the bottom that causes a decline in ALL four classification accuracies;
6. iterate steps 1∼5 until no more gene(s) can be removed. The remaining set of genes is considered the refined classifier gene set because of its small gene size and high accuracy.

### Optimization of classifier genes by clustering

Because both tree-based algorithms and SVM are supervised machine learning methods, an unsupervised clustering method was used to independently optimize the classifier genes. Clustering was performed using the K-mean clustering analysis as implemented in the WEKA toolbox. All the dendrogram trees were cut at a level so that all the 248 earthworm RNA samples were grouped into three clusters. The three pre-labelled clusters (control, RDX and TNT) served as the reference, and the three clusters derived from the dendrogram trees were compared to the reference clusters to determine matching sample numbers. The optimization of classifier genes by clustering followed the same iterative steps as described above for MC-SVM.

### Estimation of classification accuracy

Accuracy (also called true positive rate or recall) of a classifier was defined as the percentage of the dataset correctly classified by the method, i.e., number of correctly classified samples/total number of samples in the class. Due to the use of the whole dataset in feature selection, ten-fold stratified cross-validation with inner and outer loops was performed as described in [28] throughout this study to avoid sample selection bias and obtain unbiased estimates of prediction accuracies [29].

## Results

### Feature filtering by univariate statistical analysis

**Figure 2:**
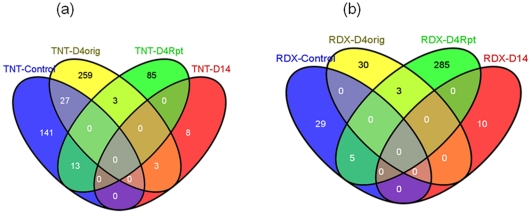
The number and overlapping of significant genes statistically inferred from class comparisons for (a) TNT and (b) RDX treatments. TNT/RDX-Control: two-class comparison between pooled controls and pooled TNT/RDX treatments; TNT/RDX-D4orig: multiple-class comparison of 4-day TNT/RDX treatments including the control group; TNT/RDX-D4Rpt: multiple-class comparison of 4-day repeat TNT/RDX treatments including the control group; TNT/RDX-D14: multiple-class comparison of 14-day TNT/RDX treatments including the control group (also see **Materials and Methods**).

Differentially expressed genes were inferred by univariate statistical analysis. At the same level of statistical stringency, the significant gene lists derived from four different comparisons for either TNT or RDX shared very few common genes (Fig. 2), suggesting different genes may be significantly altered under different conditions. To validate these results, we used ANOVA in GeneSpring GX 10 to analyze the same dataset by applying the Benjamini-Hochberg method for multiple testing corrections and a cut-off of 1.5-fold change. By allowing a variable threshold of cut-off p-value, the same amount of top significant genes can be derived from the same comparisons as we did using BRB-ArrayTools. The two sets of significant gene lists share 85∼95% common genes (data not shown), indicating a high level of statistical reproducibility. The difference in the resulting gene lists may be primarily attributed to the use of a 1.5-fold change as the cut-off level by GeneSpring. A total of 869 unique genes were obtained after combining all significantly changed gene lists from TNT- and RDX-exposures. The expression information of these 869 transcripts in all 248 earthworm samples is provided in Table S1.

### Classifier genes identified by tree-based algorithms

We used seven different tree-based machine learning algorithms to select classifier genes from the 869 statistically significant genes. Each algorithm in combination with bagging or boosting generated decision trees, separating earthworm samples into three pre-defined classes based on the expression of classifier genes. A different set of classifier genes was selected by each algorithm (Table 1). The classification accuracy varied from 75.0% for SimpleCart with boosting to 84.7% for LMT with bagging. There is a significant correlation between ROC area and accuracy (correlation coefficient = 0.94).

**Table 1:** Summary of classification results using the tree-based classification algorithms^a^.

Ensemble method	Tree-based algorithm	Accuracy (%)	ROC area
Boosting	BFTree	75.8	0.878
Boosting	J48	79.8	0.882
Boosting	LADTree	77.4	0.881
Boosting	SimpleCart	75.0	0.868
Boosting	FT	83.5	0.930
Boosting	LMT	81.8	0.936
Bagging	J48	75.4	0.868
Bagging	LADTree	75.0	0.876
Bagging	REPTree	75.0	0.870
Bagging	SimpleCart	76.2	0.855
Bagging	FT	82.7	0.937
Bagging	LMT	84.7	0.944

a A total of 354 unique classifier genes were identified.

**Figure 3:**
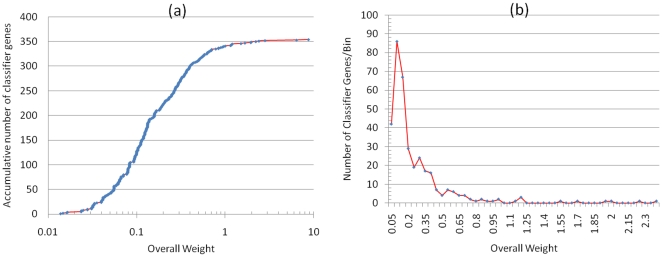
The accumulative distribution (a) and histogram (b) of the overall weight of the 354 selected classifier genes. In the histogram, the bin size is set at 0.05, and three genes with the highest overall weight of 2.81, 6.38 and 8.70, respectively, are not shown.

A total of 354 unique classifier genes were obtained after pooling classifier genes from all decision trees. Each classifier gene was then ranked by an overall weight of significance. The distribution and histogram of overall weights of these genes are shown in Fig. 3. The overall weight of 127 (or 36%) of classifier genes are below 0.1 (Fig. 3a). Only the top 43 or 14 genes had an overall weight larger than 0.5 or 1.0 (Fig. 3b), respectively. Functional annotations of the 354 genes are provided in Table S2. Over 90% of these genes have one or more strings of annotation information obtained using such bioinformatics programs as BLASTX, BLASTN, InterProScan and PIPA [30].

### Refinement of the classifier gene set using MC-SVM or clustering

**Figure 4:**
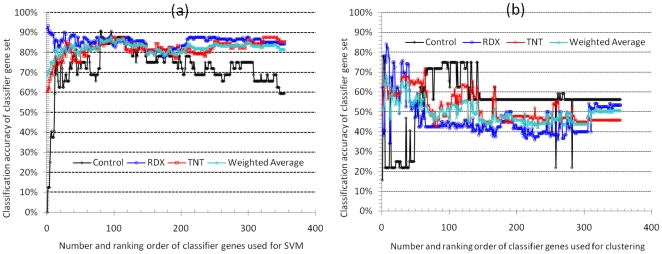
Classification accuracy of 248 earthworm samples using SVM (a) or clustering (b) with an increasing number of top ranked classifier genes. The weighted average accuracy and the accuracy for each of the three classes (control, RDX and TNT) are shown for each set of genes (1∼354 genes). Genes were added to the increasing gene set one at a time in the order of decreasing overall weight (see also **Figure 3**(a)).

**Figure 5:**
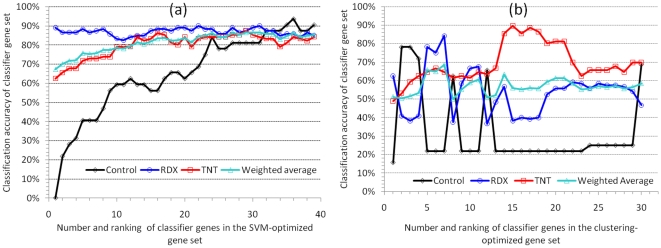
Classification accuracy of the 248 earthworm samples using an increasing number of classifier genes optimized by SVM (a) or clustering (b). The weighted average accuracy and the accuracy for each of the three classes (control, RDX and TNT) are shown for each set of genes (1∼39 genes in 5(a) or 1∼30 genes in 5(b)). One gene (the next highest ranked gene) at a time was added to the previous gene set to generate a new gene set (see also **Figure 3**(a)).

Two different algorithms, SMO and K-mean clustering, were employed to optimize the number and set of genes from the 354 ranked classifier genes. Composition of the classifier gene set had a significant influence on classification accuracy (Fig. 4). Using SMO, as few as 16 top ranked genes classified 81% of the 248 samples into correct classes (Fig. 4a). Starting at the 250^th^ gene, the inclusion of additional classifier genes not only did not improve the classification accuracy for the TNT and the RDX classes as well as the weighted average accuracy, but deteriorated the accuracy for the control class (Fig. 4a). Similarly, with the clustering approach, the top ranked 31 genes correctly clustered 66% of the samples, while addition of other genes did little, if any, to improve the accuracy of either individual classes or the weighted average (Fig. 4b). Clearly, individual classifier genes vary remarkably in its contribution to the change of classification accuracy, which also depends on the choice of machine learning algorithms. The iterative optimization process effectively removed many genes that made no or negative contribution to the classification performance. As a result, this process produced a SVM- and a clustering-optimized subset consisting of 39 and 30 genes, respectively (Fig. 5).

The first 24 genes of the SVM-optimized subset clearly played more important roles than the remaining 15 genes that only slightly improved the accuracy for the control class and changed very little the accuracy for the TNT and the RDX classes (Fig. 5a). The subset of 39 genes performed well in terms of accuracy, ROC area, and precision, with 83∼91% in accuracy and precision except for the 76% precision of the control class (Table 2). The case for the clustering-optimized subset is a bit perplexing as the accuracy of the control and the RDX classes changed in opposite directions after adding the 2^nd^, 5^th^, 7^th^, 9^th^, 11^th^ and 13^th^ genes (Fig. 5b). Nevertheless, the whole subset of 30 genes evened up the accuracy for all classes (Fig. 5b and Table 3) and gave an average of 72.5% precision for the three classes (Table 3). The sensitivity for the control class was relatively lower, especially in classification by clustering, when compared with that for the other two classes. An examination of the samples that were incorrectly clustered into the control class showed that they were mostly exposed for 4 days to the lowest three concentrations of RDX or TNT (data not shown). A plausible reason for this misclassification is that gene expression in these samples may not be significantly different from that in the controls due to low levels of contaminant. The uneven sample size might explain why the SVM precision for the control class (32 samples) is relatively lower than that for the other two classes (96 and 120).

**Table 2:** Confusion matrix showing classification results for testing datasets obtained by MC-SVM using the optimized set of 39 classifier genes.

True class (no. samples)	No. samples classified as			Accuracy (%)	ROC area
	Control	RDX	TNT		
Control (32)	29	2	1	90.6	0.938
RDX (120)	7	106	7	88.3	0.887
TNT (96)	2	14	80	83.3	0.913
Precision (%)	76.3	86.9	90.9		
Weighted average (248)	87.1 (precision)			86.7	0.904

**Table 3:** Confusion matrix showing classification results obtained by clustering^a^.

True class (no. samples)	No. samples classified as			Accuracy (%)
	Control	RDX	TNT	
Control (32)	22	1	9	68.8
RDX (120)	46	56	18	46.7
TNT (96)	21	8	67	69.8
Precision (%)	24.7	86.2	71.3	
Weighted average (248)	72.5 (precision)			58.5

a The optimized set of 30 classifier genes were used. ROC area was not computable for clustering.

### Optimized gene subset for classification

The two subsets of classifier genes optimized by SVM and clustering share 11 common genes, and the combination of these two resulted in a set of 58 unique genes that represents a refined gene set for the three-class classification. Using this gene set, we were able to build a SVM model with high performance parameters including accuracy, sensitivity and ROC area (Table 4). The classification results for both supervised SVM and unsupervised clustering are slightly less superior with the 58-gene set (Table 4) than with the 39- or the 30-gene set (Tables 2 and 3). As summarized in Table S3, 38 genes or 65.5% of the optimal gene set are among the 70 highest ranked classifier genes, 15 or 75% of the top 20 ranked genes are included in the optimal gene set, and 7 or 63.6% of the 11 genes picked by both SVM and clustering come from the top 12 ranked classifiers. These results reinforce the merit of our weight-of-significance ranking system.

**Table 4:** Confusion matrix of classification results obtained using the refined set of 58 classifier genes combined from the SVM- and the clustering-optimized gene sets.

True class (no. samples)	No. samples classified as			Accuracy (%)	ROC area
	Control	RDX	TNT		
SVM					
Control (32)	26	6	0	81.3	0.936
RDX (120)	9	100	11	83.3	0.856
TNT (96)	2	13	81	84.4	0.913
Precision (%)	70.3	84.0	88.0		
Weighted average (248)	83.8 (precision)			83.5	0.904
Clustering a					
Control (32)	22	1	9	68.8	NA
RDX (120)	48	55	17	45.8	NA
TNT (96)	22	10	64	66.7	NA
Precision (%)	23.9	83.3	71.1		
Weighted average (248)	70.9 (precision)			56.9	NA

a ROC area was not computable with clustering.

## Discussion

Microarray datasets possess an exceptionally high complexity distinguished by high feature dimension and low sample size. Like other microarray studies, the primary objective of this study was to search for an optimal or near optimal subset of genes that could be used to predict the exposure history of unknown samples. It has been proven in both theory and practice that feature selection can effectively enhance learning efficiency, increase predictive accuracy, reduce complexity of learned results, and improve the accuracy of classification models [6], [31], [32]. Although numerous supervised or unsupervised machine learning techniques have been used for feature selection and sample classification of microarray data (for reviews see [33]–[35]), classification performance appears to depend strongly on the dataset and less on the variable selection and classification methods [36]. Meanwhile, it has been demonstrated that a combined use of different classification and feature selection approaches can enhance confidence in selecting relevant genes [6], [35] and that ensemble methods such as bagging and boosting can improve classification performances [24]. These two strategies are both reflected in our ISML pipeline (Fig. 1, Table 1).

We first used the univariate statistical analysis [37] that selected 869 features/genes. These genes may represent a wide variety of transcripts that responded not only to toxicants TNT or RDX, but also likely to other environmental stresses. To further down select the features, we employed several decision-tree algorithms. A decision tree is constructed by selecting the most discriminative features/nodes for classification [35] and biomarker genes discovery [22] from microarray data. In a decision tree, the occurrence of a node (feature/gene) provides the information about the importance of the associated feature/gene [22]. The root gene has the most information gain for classification, and the other nodes genes appear in descending order of power in discrimination [38]. During the decision learning, the genes that have no discrimination capability are discarded. A total of 515 genes were eliminated from the 869 differentially expressed genes by tree-based algorithms, leaving 354 classifier genes. This represents a 59% feature reduction.

As our goal was to scale down the size of potential classifier gene set while maintaining a high discriminative power, we introduced in the ISML pipeline a new algorithm to compute and rank the overall weight of the 354 individual classifier genes based on their contribution/significance to classification. We also developed a novel optimization algorithm for iteratively removing classifier genes that had little or a negative impact on classification performance. This bottom-up removal process began with the least important gene having the lowest overall weight. We chose to eliminate those genes that only reduced the classification accuracies of all classes as well as the weighted average. This conservative approach was adopted to preserve genes that might increase the accuracy of one class but decrease that of another, like the 2^nd^, 5^th^, 7^th^, 9^th^, 11^th^ and 13^th^ genes in the clustering-optimized gene set (Fig. 5b). These genes are usually important for discriminating one particular class while confounding other classes.

SVMs are powerful classification models that have shown state-of-the-art performance on several diagnosis and prognosis tasks on biological data [24], [39]. SVM-based classification can usually achieve higher accuracy/precision on a given dataset than unsupervised algorithms. Ideally, an SVM analysis should produce a hyperplane that completely separates the feature vectors into non-overlapping groups. However, perfect separation may not be possible, or it may result in a model with so many feature vector dimensions that the model does not generalize well to other data, which is a problem commonly known as over-fitting [40]. The risk of over-fitting to the specific dataset in compensation for high accuracy/precision may render a high probability of misclassification when the trained SVM model is applied to predict unknown samples of other independent datasets. Unlike SVM, unsupervised learning algorithms can overcome this shortfall with a trade-off of less superior accuracy/precision. Our ISML pipeline adopted a compromise between these two approaches. Although the effectiveness, efficiency and superiority of this approach has to go through more stringent validation and testing, our results indicate that the final combined gene set produced nearly as good classification outcome as the two separately optimized gene subsets. This combined gene set need to be tested in field samples where exposure history including species and concentration of contaminants as well as exposure length is often unknown. Currently, a field soil study is undertaken to validate this optimal gene set, where lab-cultured mature earthworms are exposed in field soils primarily contaminated with TNT or RDX.

Classification accuracy was evaluated in this study on a sole basis of the pre-defined exposure history, that is, each sample was labelled with *a priori* class corresponding to the chemical it had been exposed to, disregarding the differences in soil concentration of TNT or RDX. The accuracy of biological classification can be impaired for soils containing low toxicant concentrations which may not induce gene expression effects significant enough to distinguish exposed animals from the controls. This might contribute partly to the lower accuracy obtained from clustering than from SVM. It is desirable to define a threshold such as the lowest observable effect concentration expressed as the toxicant concentration in soil or animals (body burden). We prefer body burden as an exposure measure over soil concentration due to the often heterogeneous distribution of toxicants in soil. This way, animals with a tissue concentration below the threshold can be grouped/pre-defined together with unexposed control animals, which potentially benefits clustering more than SVM.

To define a sensitive threshold, one can measure disease-related biological endpoints that are presumably more sensitive than the mortality and growth endpoints in short-term exposures of 4 or 14 days. Alternatively, one can measure toxicity-related phenotypic (e.g., biochemical, physiological, or pathological) endpoints if a more toxicological meaningful discrimination is desired. The SVM classification model for exposure classification in the output of the ISML pipeline can be conveniently converted into a disease or toxicity diagnosis model.

Another confounding factor that affects classification accuracy is that vulnerability and susceptibility vary from one animal to another, which may be caused by many factors such as genetic make-up, age, and physiological status. We believe that the diagnosis or prediction accuracy of unknown samples can be greatly improved if gene expression profiles of biologically well-characterized, pre-defined animals are used as the training dataset, just like in cancer microarray studies.

Among the 58 optimized genes, 93% genes exhibited toxicant-specific gene expression alterations, that is, 32 genes responded specifically to TNT, 22 to RDX, and only 4 to both chemicals (Table S3). Forty-two genes (72%) have meaningful annotation with a wide range of biological functions spanning from antioxidant response (COX4 and NADH-coenzyme Q reductase) to spermatogenesis (evcp-2) and GABA receptor modulator (DBI, also known as Acyl-CoA-binding protein or ACBP). Three of the top 10 ranked genes, PTB, DBI and SOD, have previously been shown being altered by TNT [7] or RDX [8]. Two probes targeting two highly similar transcripts coding for evcp-2, a gene expressed specifically in the anterior segments of sexually mature earthworms [41], take the 10^th^ and the 28^th^ positions on the optimal gene list, suggesting that both TNT and RDX may affect spermatogenesis. On the list, there are also several stress-responding genes such as HSP70 (#13 & #41) [42] as well as cancer-related genes such as TCTP (#57) [43]. It is worth noting that six genes, PTB (#1) [44], DDX46 (#3) [45], EF2 (#15 & #34) [46], hnRNP K (#16) [47], and eRF1 (# 26) [48] are all involved in mRNA splicing or processing and RNA translation initiation or termination, indicating alteration of mRNA secondary structure and protein synthesis may be targeted by both TNT and RDX. More work should be devoted to exploring biological functions and interactions of the 58 genes that may lead or be linked to toxicological effects or biochemical endpoints.

This study addresses a sophisticated issue of discovering and optimizing classifier gene sets in environmentally relevant animal models. Although a perfect or the best solution to it is yet to be found, we have demonstrated that the ISML pipeline can reduce the dimensionality of microarray datasets, identify and rank classifier genes, generate a small set of classifier genes, produce an SVM classification model with high accuracy, and select a small group of biomarker candidate genes for biological validation. This approach can also be applied to discover diagnostic biomarker genes exhibiting toxicity- or disease-dependent response in environmental species from fish and springtail to water flea and earthworm.

We report here some preliminary results of a much larger effort. Our future work include: (1) compare the performance of the ISML approach with that of other popular and existing feature selection techniques such as SVM-RFE (SVM Recursive Feature Elimination), CFS (Correlation based Feature Selection) and χ^2^ using the earthworm dataset and other microarray datasets; (2) validate the final 58- gene set using other experimental methods such as real-time quantitative PCR, (3) further test the classifiers in field samples; (4) identify TNT/RDX concentration-related classifier genes; and (5) validate the biochemical outcome regulated by the biomarker candidate genes. We believe that these consorted efforts will lead us to discovery of novel biomarker genes useful for environmental risk assessment.

## Supporting Information

Table S1 Treatment information of 248 earthworm samples and expression data of the 869 differentially expressed genes.
(3.97 MB XLS)

Table S2 Array oligo probe ID, target gene ID, probe and their target gene sequences, overall weight, functional annotation, and treatment(s) that altered the gene expression.
(0.32 MB XLS)

Table S3 The optimized set of 58 classifier genes as an output of the ISML pipeline
(0.12 MB DOC)
