# Supplementary material for: Identification and Optimization of Classifier Genes from Multi-Class Earthworm Microarray Dataset
Source: PLoS One. 2010 Oct 28;5(10):e13715. doi: 10.1371/journal.pone.0013715 (PMC2965664; doi:10.1371/journal.pone.0013715)
Supplement: Table S3 — The optimized set of 58 classifier genes as an output of the ISML pipeline (0.12 MB DOC) [file pone.0013715.s003.doc]

**Table S3 -** The optimized set of 58 classifier genes as an output of the ISML pipeline

| Gene order | Probe name ^a^ | Overall weight | Rank ^b^ | Picked  By | Expression altered by | Target gene annotation |
| --- | --- | --- | --- | --- | --- | --- |
| 1 | TA2-091233 | 8.70 | 1 | Both | RDX | Polypyrimidine tract binding (PTB) protein |
| 2 | TA1-023824 | 6.38 | 2 | Both | TNT | NADH-coenzyme Q reductase |
| 3 | TA1-204280 | 2.81 | 3 | Clustering | RDX | DEAD box polypeptide 46 (DDX46) |
| 4 | TA1-012917 | 2.40 | 4 | Both | TNT | Unavailable |
| 5 | TA2-092252 | 2.21 | 5 | Both | RDX | Diazepam binding inhibitor (DBI)-like protein |
| 6 | TA1-022179 | 1.96 | 6 | Both | RDX | Signal-peptide |
| 7 | TA1-200771 | 1.94 | 7 | Both | TNT | Superoxide dismutase (SOD) |
| 8 | TA1-003377 | 1.50 | 9 | SVM | RDX | Unavailable |
| 9 | TA2-113782 | 1.20 | 10 | SVM | Both | Signal-peptide |
| 10 | TA2-139945 | 1.17 | 12 | Both | TNT | Earthworm valosine containing peptide-2 (evcp-2) |
| 11 | TA2-210040 | 1.14 | 13 | SVM | TNT | Unavailable |
| 12 | TA1-173733 | 1.00 | 14 | Clustering | RDX | Signal recognition particle |
| 13 | TA2-206312 | 0.96 | 15 | Clustering | TNT | Heat shock protein 70 (HSP70) |
| 14 | TA1-200094 | 0.84 | 19 | Clustering | RDX | Serine/Threonine protein phosphatise |
| 15 | TA1-084360 | 0.78 | 20 | SVM | TNT | Translational elongation factor 2 (EF2) |
| 16 | TA2-056405 | 0.70 | 21 | SVM | TNT | Heterogeneous nuclear ribonucleoprotein (hnRNP) K |
| 17 | TA2-029918 | 0.66 | 25 | SVM | Both | Unknown |
| 18 | TA1-189015 | 0.65 | 26 | SVM | RDX | 60S acidic ribosomal protein P2 |
| 19 | TA2-153080 | 0.62 | 29 | SVM | RDX | Electron transfer flavoprotein (ETF) β-subunit |
| 20 | TA2-135639 | 0.58 | 32 | SVM | Both | Unavailable |
| 21 | TA1-056351 | 0.56 | 34 | Clustering | TNT | Unavailable |
| 22 | TA1-167854 | 0.55 | 35 | SVM | RDX | Unknown |
| 23 | TA2-099898 | 0.55 | 36 | Clustering | TNT | Presenilin |
| 24 | TA2-005815 | 0.53 | 38 | SVM | TNT | Unknown |
| 25 | TA1-065695 | 0.52 | 39 | SVM | TNT | Unknown |
| 26 | TA2-088504 | 0.51 | 41 | SVM | TNT | Eukaryotic release factor 1 (eRF1) |
| 27 | TA1-020439 | 0.50 | 42 | Clustering | RDX | Unknown |
| 28 | TA1-194525 | 0.50 | 43 | Both | Both | Valosine containing peptide-2 (evcp-2) |
| 29 | TA2-146992 | 0.45 | 47 | SVM | RDX | ATP synthase 9 mitochondrial |
| 30 | TA2-058573 | 0.44 | 48 | Clustering | TNT | Arginine/Serine-rich splicing factor |
| 31 | TA1-086892 | 0.42 | 50 | Clustering | TNT | Unavailable |
| 32 | TA1-030037 | 0.42 | 51 | SVM | RDX | S10_Plectin |
| 33 | TA2-058673 | 0.41 | 52 | SVM | TNT | Unavailable |
| 34 | TA2-006089 | 0.40 | 55 | SVM | TNT | Translational elongation factor 2 (EF2) |
| 35 | TA1-213621 | 0.37 | 63 | Both | TNT | Vacuolar ATP synthase proteolipid subunit |
| 36 | TA1-058331 | 0.36 | 67 | SVM | TNT | Titin |
| 37 | TA1-095832 | 0.36 | 68 | Clustering | RDX | Unknown |
| 38 | TA2-144740 | 0.35 | 70 | SVM | RDX | 26S proteasome complex subunit DSS1 |
| 39 | TA1-183759 | 0.32 | 78 | Both | RDX | 26S proteasome regulatory subunit RPN1 |
| 40 | TA1-058194 | 0.32 | 80 | SVM | TNT | Unknown |
| 41 | TA1-026013 | 0.31 | 83 | SVM | TNT | Heat shock protein 70 (HSP70) |
| 42 | TA1-017276 | 0.31 | 85 | SVM | TNT | RNA-binding domain, RBD |
| 43 | TA2-203946 | 0.31 | 86 | SVM | TNT | Peptidase (mitochondrial processing) β isoform 2 |
| 44 | TA1-153822 | 0.30 | 88 | Clustering | TNT | Pyruvate kinase |
| 45 | TA1-184000 | 0.30 | 89 | Clustering | TNT | NADPH FAD oxidoreductase |
| 46 | TA1-193191 | 0.27 | 102 | Clustering | RDX | Cytochrome c oxidase subunit IV (COX4) |
| 47 | TA1-118706 | 0.27 | 104 | SVM | TNT | Oxidoreductase |
| 48 | TA2-119638 | 0.25 | 116 | SVM | TNT | Unavailable |
| 49 | TA2-118209 | 0.23 | 121 | Clustering | TNT | Unavailable |
| 50 | TA2-081772 | 0.20 | 132 | Clustering | TNT | NADH-coenzyme Q reductase |
| 51 | TA1-208104 | 0.14 | 163 | SVM | TNT | Lipocalins |
| 52 | TA1-118589 | 0.11 | 207 | SVM | RDX | 40S ribosomal protein S10 |
| 53 | TA2-031808 | 0.10 | 225 | Clustering | RDX | Biogenesis of lysosome-related organelles complex-1 subunit |
| 54 | TA1-123240 | 0.09 | 246 | Clustering | RDX | RNA-binding protein |
| 55 | TA2-090604 | 0.08 | 254 | Clustering | TNT | Arginine/Serine-rich coiled-coil 2 isoform 2 |
| 56 | TA1-008487 | 0.08 | 265 | Clustering | TNT | Cytochrome C1 |
| 57 | TA2-118601 | 0.08 | 266 | Both | RDX | Translationally-controlled tumor protein homolog (TCTP) |
| 58 | TA2-095503 | 0.04 | 319 | SVM | RDX | 60S ribosomal protein RPL36A |

^a^ See Tables S1 and S2 for more sequence and annotation information of the probes and their target genes.

^b^ Rank indicates the weight-of-significance ranking among the 354 classifier genes.
